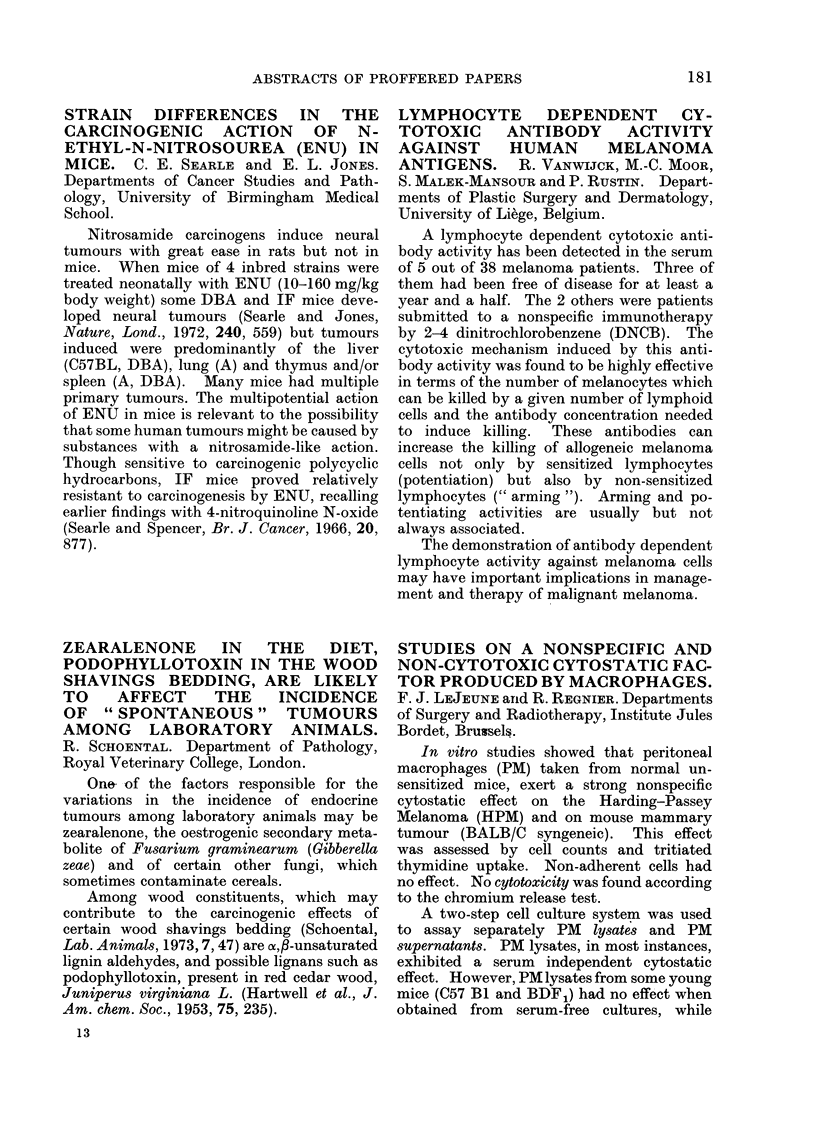# Proceedings: Zearalenone in the diet, podophyllotoxin in the wood shavings bedding, are likely to affect the incidence of "spontaneous" tumours among laboratory animals.

**DOI:** 10.1038/bjc.1974.159

**Published:** 1974-08

**Authors:** R. Schoental


**ZEARALENONE IN THE DIET, PODOPHYLLOTOXIN IN THE WOOD SHAVINGS BEDDING, ARE LIKELY TO AFFECT THE INCIDENCE OF " SPONTANEOUS " TUMOURS AMONG LABORATORY ANIMALS.** R. SCHOENTAL. Department of Pathology, Royal Veterinary College, London.

One of the factors responsible for the variations in the incidence of endocrine tumours among laboratory animals may be zearalenone, the oestrogenic secondary metabolite of *Fusarium graminearum* (*Gibberella zeae*) and of certain other fungi, which sometimes contaminate cereals.

Among wood constituents, which may contribute to the carcinogenic effects of certain wood shavings bedding (Schoental, *Lab. Animals*, 1973, 7, 47) are $\alpha,\beta$-unsaturated lignin aldehydes, and possible lignans such as podophyllotoxin, present in red cedar wood, *Juniperus virginiana L.* (Hartwell *et al.*, *J. Am. chem. Soc.*, 1953, **75**, 235).